# Microbial Biofilm Reduces the Strength Reliability of 3D-Printed Appliance Resins

**DOI:** 10.3390/dj14080487

**Published:** 2026-08-05

**Authors:** Watt Sook May, Ng Zicong, Vinicius Rosa, Kelvin Weng Chiong Foong

**Affiliations:** Faculty of Dentistry, National University of Singapore, 9 Lower Kent Ridge Road, Singapore 119085, Singapore; maywatt@u.nus.edu (W.S.M.); e0235139@u.nus.edu (N.Z.); kenfwc@nus.edu.sg (K.W.C.F.)

**Keywords:** acrylic resins, printing, three-dimensional, flexural strength, materials testing, biofilms

## Abstract

**Background/Objectives**: Three-dimensionally (3D) printed resins are increasingly used for removable orthodontic appliances, but how their mechanical reliability changes in the oral environment is unclear. This study evaluated the effect of bio-ageing environment and material type on the biaxial flexural strength and Weibull reliability of one conventional acrylic resin (Orthocryl, OC) and two 3D-printed photopolymer resins (BioMed Clear, BMC; and KeySplint Hard, KH). The Weibull modulus (m) reflects how consistent, or predictable, the strength is among specimens, independent of its average value. **Methods:** Disc specimens (n = 32) were aged for 24 h at 37 °C in pooled human saliva, a *Streptococcus mutans* biofilm, or water, and the biaxial flexural strength was measured by the piston-on-three-balls method (1 mm/min). Data were analysed with two- and three-parameter Weibull statistics to estimate the Weibull modulus (m), characteristic strength (σ_0_), strength at 5% failure probability (σ_5_%), and threshold strength (σ_μ_), with model selection guided by the Akaike Information Criterion. **Results:** Biofilm exposure lowered m while σ_0_ was preserved: m fell from 9.96 (saliva) and 7.98 (water) to 1.95 for OC, and from 17.61 and 13.01 to 2.62 for BMC, whereas σ_0_ stayed near 55 MPa (OC) and 64 MPa (BMC). Under biofilm the modulus of all three converged to low values (1.4–2.6), abolishing the differences among materials; OC and BMC required a three-parameter model only after biofilm (σ_u_ ≈ 41 MPa), whereas KH required it in every environment (σ_u_ = 28.9–38.7 MPa). **Conclusions:** Biofilm ageing mainly reduced strength predictability, while characteristic strength was largely preserved. This indicates that flexural strength alone may be insufficient to qualify resins for removable appliances, and that printed resins studied here are not inherently superior or inferior to conventional acrylic.

## 1. Introduction

Retainers and occlusal splints are used for tooth position maintenance, dentition protection, and management of parafunctional or occlusal conditions. Acrylic resins and thermoformed polymers are widely used to fabricate such appliances, but their performance can be affected by water sorption, surface degradation, wear, fracture, and microbial adhesion. Thermoformed retainers have manufacturing limitations like roughened surfaces that may promote biofilm retention and material deterioration [1].

Polymeric dental appliances are frequently exposed to saliva and microbial biofilms (bioaging) that can compromise the materials’ performance. The exposure of resin-based specimens to *S. mutans* reduced specimens’ flexural strength and roughness compared to those exposed to bacteria-free medium or distilled water [2,3,4]. The surfaces in contact with biofilms are exposed to acidic metabolites, enzymes, and water, promoting plasticization, hydrolysis, leaching, surface roughening, and polymer weakening [5,6]. Similar concerns exist in polymer specimens subjected to thermomechanical cycling or acidic conditions, which increase roughness and hydrophilicity, reducing hardness and flexural properties [4,7]. These findings suggest saliva, pH, water uptake, and biofilms affect surface and mechanical properties. Such biofilm-mediated degradation is a recognised cause of biodeterioration in polymeric materials and can involve multi-species communities as well as single species [5,8]. Thus, understanding the extent of biologically driven degradation is essential for improving materials and manufacturing technologies.

Direct 3D printing is increasingly used to fabricate retainers and occlusal splints because it allows greater design control. Exposure of 3D-printing resins and such devices to biologically relevant microenvironments reduces their mechanical properties unevenly. For instance, the flexural strength of appliance polymers declines to a material-specific extent; after 90 days of water storage, a 3D-printed splint resin lost about 75% of its strength, whereas conventional injection-molded acrylics were less affected, losing about 20 to 33% [9]. Likewise, thermomechanical cycling roughened heat-cured acrylic and milled poly(methyl methacrylate) (PMMA) more than 3D-printed PMMA (ΔR_a_ of 3.3 and 3.0 µm vs. 1.1 µm) and reduced the flexural strength of heat-cured acrylic by about one-third (from ≈105 to ≈70 MPa), whereas the 3D-printed resin did not weaken (≈105 to ≈160 MPa) [10]. However, water storage and thermomechanical cycling do not reproduce the salivary and microbial biofilm challenges encountered intraorally. Under oral-like acidic or biofilm-related conditions, 3D-printed resins may become rougher, more hydrophilic, and softer, while prolonged exposure to artificial saliva can further increase surface roughness and compromise their biomechanical properties [11,12]. This variability is also seen for other additively manufactured materials: printed and milled zirconia can reach a similar mean flexural strength, yet printed zirconia shows a lower Weibull modulus and thus less predictable performance [13,14]. Across these protocols, artificial aging alters both the mechanical and physical properties of appliance polymers in a material-specific manner; its effect on mechanical reliability under biologically relevant aging remains to be established.

Fracture strength is commonly used to estimate the structural performance of materials. Nevertheless, these values do not directly predict structural performance, as strength is a contingent property rather than an inherent one [15]. To describe the variability in fracture strength measurements of brittle materials, Weibull statistical analysis can be used. This analysis relates to fracture probability and can assess material reliability, such as the stress needed to fracture a certain percentage of specimens [15]. The two-parameter Weibull distribution relies on the Weibull modulus and characteristic strength. The Weibull modulus (m) is a dimensionless parameter unique to each material, indicating the degree of strength variation or asymmetry in the strength distribution due to microstructural flaws [15,16,17]. A lower modulus reflects greater variability and reduced reliability, whereas a higher modulus indicates a narrower distribution of strength and a more uniform flaw population, and therefore greater reliability and a lower probability of failure [15,16]. Clinically, two resins with the same average strength are not equivalent: the one with the lower modulus produces more appliances that fracture at unexpectedly low loads, so reliability is a direct indicator of expected service life. The two-parameter model, however, implicitly assumes a threshold strength of zero, so that failure may occur at any non-zero stress [15]. This assumption can misrepresent the lower tail of the strength distribution when the data deviate from linearity on a Weibull plot. Therefore, alternative distributions that introduce a location parameter, the threshold strength, below which failure is not expected, may be required in cases where the two-parameter distribution is not enough.

Currently, it is uncertain whether saliva and biofilms affect the strength and reliability of 3D-printed resins differently from PMMA. This is critical because long-term performance depends on the predictability of strength under oral bio-aging conditions. Therefore, this study aimed to evaluate the effects of material type and bio-aging environment on the biaxial flexural strength and Weibull parameters of two 3D-printed resins compared with a conventional acrylic resin control. The null hypothesis was that material type and aging medium would not significantly affect biaxial flexural strength, Weibull modulus, or characteristic strength.

## 2. Materials and Methods

### 2.1. Materials and Specimen Preparation

One conventional acrylic resin, Orthocryl^®^ (OC; Dentaurum, Ispringen, Germany), and two 3D-printed photopolymer resins, BioMed Clear Resin (BMC; Formlabs, Somerville, MA, USA) and KeySplint Hard (KH; Keystone Industries, Gibbstown, FL, USA), were evaluated. These materials were selected to represent the options used for removable appliances: a conventional autopolymerised acrylic (OC, a methyl-methacrylate powder/liquid system) and two methacrylate-based photopolymer resins marketed for 3D-printed retainers and occlusal splints (BMC and KH), all indicated for comparable clinical uses. Disc-shaped specimens were prepared for each material and allocated to three bio-aging conditions: *Streptococcus mutans* biofilm, pooled human saliva, or deionized water (n = 32 per material/condition).

OC specimens were fabricated by applying alternating increments of polymer powder and liquid monomer into the mould according to the manufacturer’s instructions. For BMC and KH, disc specimens were digitally designed using Asiga Composer (Asiga, Sydney, Australia) with target dimensions of 12 mm in diameter and 1.2 mm in thickness. Specimens were printed using an Asiga Max UV digital light processing printer (Asiga, Sydney, Australia) at an 80° build angle, with support structures positioned around the disc margins and the manufacturer-recommended layer thickness for each resin. Printed specimens were washed in 96% isopropyl alcohol for 5 min and post-cured for 30 min at 60 °C under ultraviolet light (375 nm).

All specimens were finished to standardize dimensions and surface condition before ageing. Specimens were ground using a sequence of P600, P800, and P1200 silicon carbide abrasive papers (CarbiMet, Buehler, Lake Bluff, IL, USA), and one surface was mirror-polished using an automated polishing machine (Ecomet 30, Buehler) until a final thickness of 1.0 ± 0.1 mm was obtained.

*S. mutans* (UA159, ATCC) was subcultured on brain heart infusion agar (BHI; Sigma-Aldrich, St. Louis, MO, USA). Colonies were transferred to BHI broth and incubated for 16 h at 37 °C in an orbital shaker at 80 rpm. The bacterial suspension was centrifuged at 4000 rpm for 5 min at 4 °C, and the pellet was resuspended in fresh BHI broth. The optical density was adjusted to 0.6–0.7 at 600 nm, corresponding to approximately 107 cells/mL. The *S. mutans* culture and biofilm-growth conditions followed our previously established protocol [18]. Commercially available pooled human saliva (approval NUS-IRB-2023-875) was obtained from Medix Biochemica (St. Louis, MO, USA).

For ageing, each specimen was placed individually in a well of a sterile, flat-bottomed 24-well polystyrene plate (Greiner Bio-One, Singapore). Specimens were immersed in 1 mL of OD-adjusted *S. mutans* suspension, pooled human saliva, or deionized water. Plates were incubated for 24 h at 37 °C in 5% CO_2_. After ageing, specimens were disinfected with alcohol spray and washed once with 1 mL of deionized water. Biofilm formation on the specimens was confirmed by visual inspection after the incubation period.

### 2.2. Biaxial Flexural Strength Testing

Biaxial flexural strength was determined using a piston-on-three-balls setup according to ISO 6872:2015 [19]. Specimens were tested in a universal testing machine (AGS-500G, Shimadzu, Tokyo, Japan) at crosshead speed of 1 mm/min until fracture.

Biaxial flexural strength (σ) was calculated from the fracture load using Equations (1)–(3), where P is the fracture load, b is specimen thickness, ν is Poisson’s ratio, r_1_ is the radius of the support circle, r_2_ is the radius of the loaded area, and r_3_ is the specimen radius. A Poisson’s ratio of 0.36 was used for the resin materials.

(1) $$\sigma =\frac{-0.237P(X-Y)}{b^{2}}$$

(2) $$X=\left(1+v\right)ln{\left(\frac{r_{2}}{r_{3}}\right)}^{2}+\left(\frac{1-v}{2}\right){\left(\frac{r_{2}}{r_{3}}\right)}^{2}$$

(3) $$Y=\left(1+v\right)\left(1+ln{\left(\frac{r_{1}}{r_{3}}\right)}^{2}\right)+\left(1-v\right){\left(\frac{r_{1}}{r_{3}}\right)}^{2}$$

### 2.3. Weibull Analysis

For each material/condition, fracture strength values were ranked in ascending order and assigned a cumulative probability of failure for graphical representation. The two-parameter Weibull distribution was fitted using maximum likelihood estimation according to Equation (4), where Pf is the probability of failure, σ is the fracture strength, σ_0_ is the characteristic strength corresponding to 63.2% probability of failure, and m is the Weibull modulus. The Weibull modulus was used as an indicator of strength variability and reliability, with higher m values indicating a narrower strength distribution.

(4) $$Pf\left(\sigma \right)=1-\exp\left(-{\left(\sigma /\sigma_{0}\right)}^{\;m}\right)$$

To account for datasets that deviated from a conventional linear Weibull trend, a generalized three-parameter Weibull model was also evaluated (Equation (5)), where σ_μ_ is the lower-strength boundary below which failure is not expected under the fitted distribution. For the two-parameter model, σ_μ_ was fixed at zero. The parameters m, σ_0_, and σ_μ_ were estimated by maximum likelihood fitting. The strength corresponding to 5% probability of failure (σ_5_%) was calculated from the fitted Weibull distribution as a clinically relevant lower-bound strength estimate.

(5) $$Pf\left(\sigma \right)=1-\exp\left(-{\left(\frac{\sigma -\sigma_{\mu }}{\sigma_{0}}\right)}^{m}\right)$$

Selection between the two- and three-parameter models was made objectively rather than by visual inspection of the probability plots. For each dataset, the Akaike Information Criterion (AIC) was computed for the two-parameter fit. For the three-parameter fit, the threshold strength (σ_μ_) was initialized at the 5th percentile of the strength data and refined via a golden-section search to minimize the AIC. The improvement afforded by the additional parameter was quantified as ΔAIC = AIC_2p_ − AIC_3p_, and the three-parameter model was adopted only when it produced a strictly lower AIC than the two-parameter model (ΔAIC > 0); otherwise, the two-parameter model (σ_μ_ = 0) was retained. Approximately 30 specimens provide stable two-parameter Weibull estimates, with additional specimens adding little precision [20]; the 32 specimens per group used here meet this recommendation.

To assess the stability of the fitted Weibull parameters, bootstrap resampling was performed for each material/condition. From each original dataset, 2000 bootstrap samples were generated by resampling the fracture strength values with replacement. For each bootstrap sample, the Weibull parameters were re-estimated using the same maximum likelihood procedure, with σ_μ_ fixed at zero for the two-parameter model and optimized using the same AIC-based procedure for the three-parameter model. The bootstrap estimates were used to evaluate the robustness of m, σ_0_, σ_μ_, and σ_5_% and to obtain their 95% confidence intervals (Table 1).

## 3. Results

### 3.1. Strength–Probability Plots and Weibull Model Fitting

**Figure 1:**
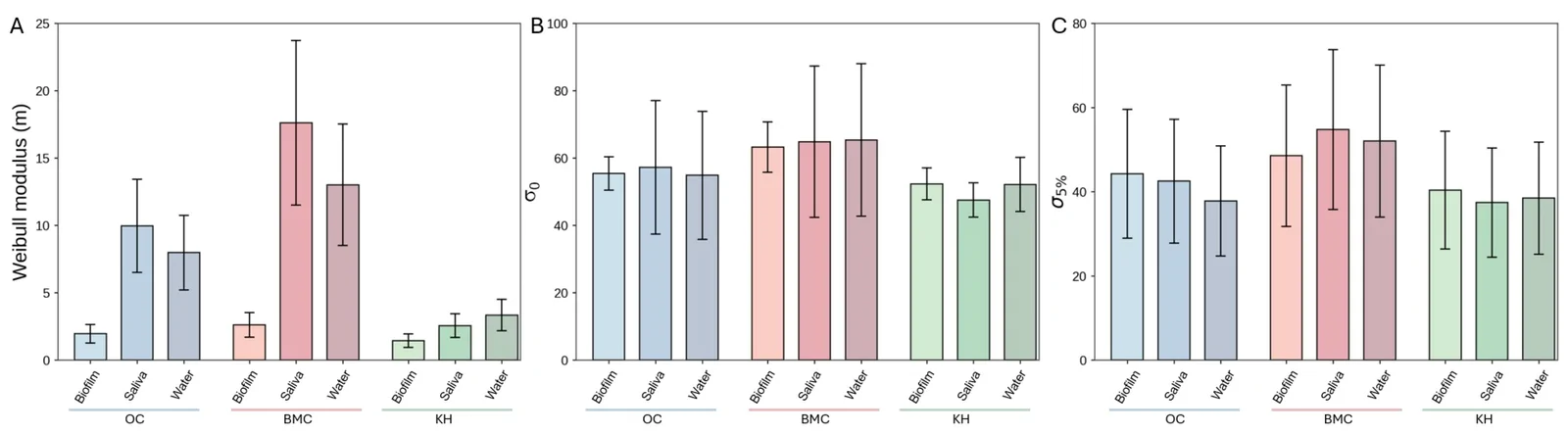
Weibull reliability parameters after bio-ageing. (**A**) Weibull modulus (m), (**B**) characteristic strength (σ_0_), and (**C**) strength at 5% fracture probability (σ_5_%) estimated from the selected Weibull model for OC, BMC, and KH after 24 h in *S. mutans* biofilm, pooled human saliva, or water. Each material is identified by a distinct colour. Within each material, the biofilm group generally showed the lowest m, indicating greater strength variability after microbial challenge. BMC generally showed the highest reliability and strength parameters, whereas OC and KH showed lower and partially overlapping values. σ_0_ remained relatively stable across media for OC and BMC, whereas σ_5_% showed no consistent medium-dependent trend. Error bars indicate 95% confidence intervals.

**Figure 2:**
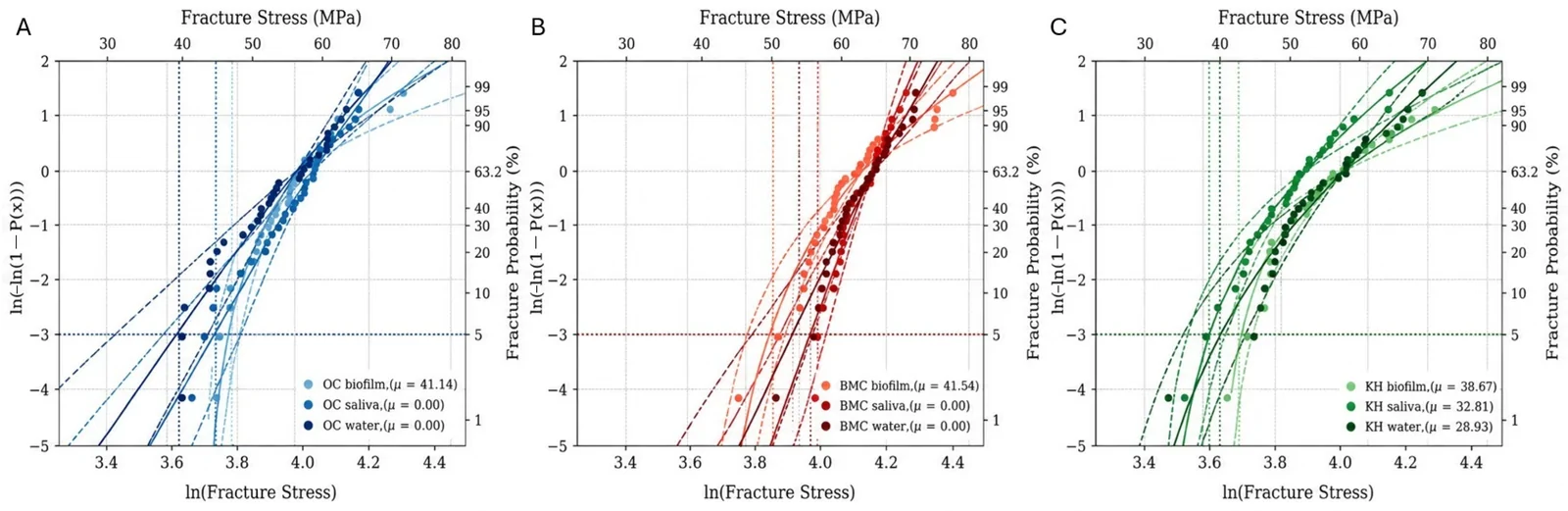
Material-specific Weibull strength–probability plots. Generalized Weibull strength–probability plots for (**A**) OC, (**B**) BMC, and (**C**) KH in biofilm, saliva, and water (threshold strength σ_μ_, shown as μ in the panels; σ_μ_ = 0 denotes a two-parameter fit, whereas σ_μ_ > 0 denotes a three-parameter fit with a lower-strength boundary). All three panels use the same axes to allow direct comparison. Horizontal dotted lines indicate 5% fracture probability, and vertical dotted lines indicate the corresponding σ_5_% values. For OC and BMC, saliva and water followed a two-parameter trend, whereas biofilm exposure and all KH groups were better described by non-zero σ_u_ values.

**Figure 3:**
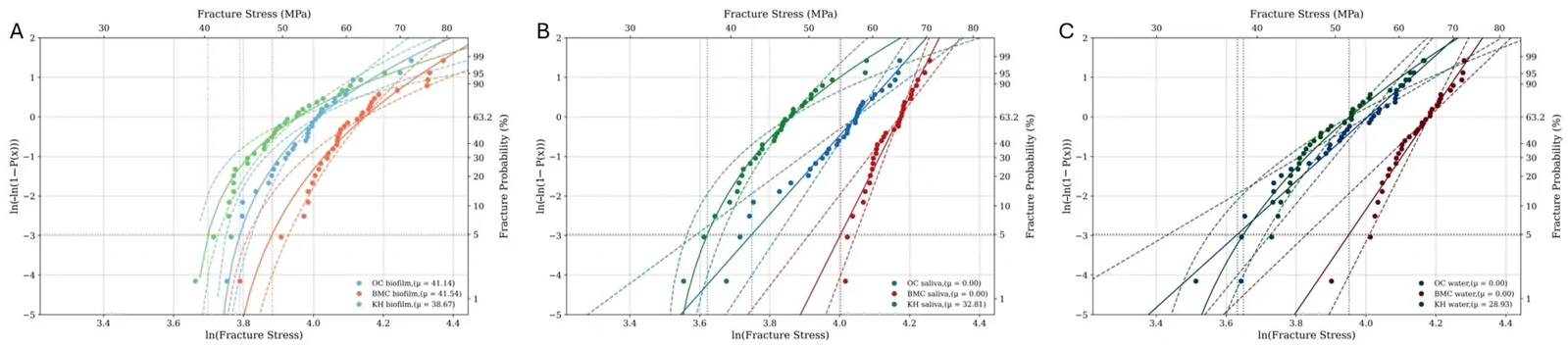
Ageing-condition-specific Weibull strength–probability plots. Generalized Weibull strength–probability plots comparing OC, BMC, and KH within (**A**) biofilm, (**B**) saliva, and (**C**) water. Axes, symbols, fitted curves, σ_u_ values, and σ_5_% indicators are as described in Figure 2.

The two-parameter Weibull model was initially fitted to all groups as the standard approach for strength reliability analysis. OC saliva, OC water, BMC saliva, and BMC water were adequately described by the two-parameter model, with σ_μ_ = 0. In contrast, OC biofilm, BMC biofilm, and all KH groups were better described by the three-parameter model, with non-zero σ_μ_ values ranging from 28.9 to 41.5 MPa. Thus, biofilm exposure altered the strength distribution of OC and BMC, whereas KH showed generalized Weibull behavior across all ageing conditions (Figure 1, Figure 2 and Figure 3). All fitted parameters, with their 95% confidence intervals, are summarised in Table 1.

### 3.2. Effect of Ageing Environment on Weibull Parameters

The ageing environment mainly affected the Weibull modulus, indicating changes in strength variability rather than a uniform reduction in strength (Figure 1A, Table 1). Within each material, biofilm exposure produced the lowest m value. This effect was most evident for OC, where m decreased from 9.9 in saliva and 7.9 in water to 1.95 after biofilm exposure (about 80% lower than saliva), and for BMC, where m decreased from 17.6 in saliva and 13.0 in water to 2.62 after biofilm exposure (about 85% lower); for both materials the 95% confidence intervals of the saliva and water groups did not overlap those of the biofilm groups. KH showed consistently low m values across all conditions, ranging from 1.43 to 3.3.

The characteristic strength showed smaller medium-dependent changes than the Weibull modulus (Figure 1B, Table 1). OC maintained similar σ_0_ values across ageing conditions, with overlapping 95% confidence intervals, whereas BMC consistently showed the highest σ_0_ values (about 64–65 MPa, roughly 15% higher than OC and 20% higher than KH). KH showed lower σ_0_ values overall. The strength at 5% probability of failure (σ_5_%) also showed no consistent medium-dependent pattern, although BMC generally maintained higher σ_5_% values than OC and KH (Figure 1C). Overall, biofilm exposure had a stronger effect on m than on σ_0_ or σ_5_%.

### 3.3. Material- and Condition-Specific Weibull Responses

The material-specific plots showed distinct responses to ageing (Figure 2). For OC and BMC, saliva and water produced approximately linear Weibull distributions, whereas biofilm exposure shifted the response toward a generalized Weibull distribution and markedly reduced m. For KH, all ageing conditions required non-zero σ_μ_ values and showed low m values, indicating a broader and less predictable strength distribution regardless of ageing medium.

When materials were compared within each ageing condition, BMC showed the most favorable overall strength profile, with higher σ_0_ and σ_5_% values than OC and KH (Figure 3). Under biofilm ageing, however, all materials showed reduced reliability and were better described by generalized Weibull fits. Under saliva and water ageing, BMC remained right-shifted relative to OC and KH, while OC and KH showed greater overlap in the lower-strength region.

**Figure 4:**
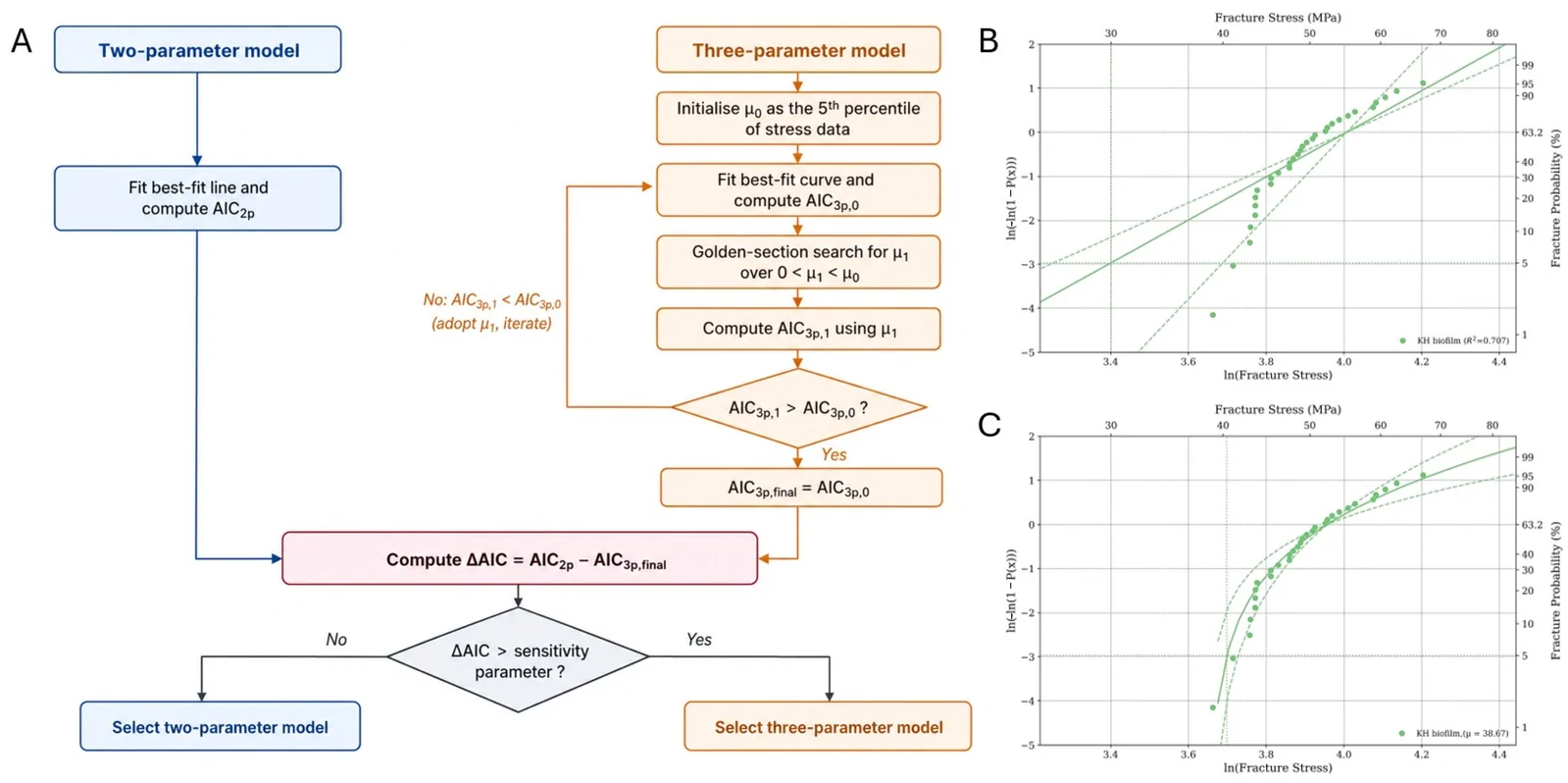
Workflow for selecting between two- and three-parameter Weibull models. (**A**) Decision algorithm: the Akaike Information Criterion (AIC) is computed for the two-parameter fit and for the three-parameter fit, whose threshold strength (σ_μ_, shown as μ in the panels) is initialised at the 5th percentile of the strength data and refined by a golden-section search; the three-parameter model is adopted only when it gives a strictly lower AIC (ΔAIC > 0). (**B**,**C**) Worked example for KH in biofilm fitted with (**B**) the two-parameter model (forced linear fit, R^2^ = 0.707) and (**C**) the three-parameter model (σ_μ_ = 38.7 MPa).

Model selection was confirmed using the AIC-based criterion (Figure 4A). KH biofilm illustrates the effect of model choice: the two-parameter model forced a linear fit through a non-linear distribution (R^2^ = 0.707; Figure 4B), whereas the three-parameter model, with σ_u_ = 38.7 MPa, better followed the lower-strength region and yielded a higher σ_5_% (Figure 4C).

## 4. Discussion

Three-dimensionally (3D) printed resins are increasingly used for removable orthodontic appliances, but how their mechanical reliability changes in the oral environment is unclear. Mechanical reliability is clinically relevant because failure risk is governed not only by average strength, but also by the consistency of strength among specimens. A material with similar characteristic strength may still be less reliable if ageing increases the variability of its flaw population. This distinction was captured by the Weibull analysis and led to partial rejection of the null hypothesis, since both material type and ageing environment affected the reliability parameters. Biofilm exposure reduced the Weibull modulus of OC and BMC, whereas the characteristic strength was largely preserved (Figure 1). This effect was particularly evident for BMC, in which m fell from 13.0 in water to 2.62 after biofilm exposure, while σ_0_ remained near 64 MPa. A reduction in m without a corresponding decrease in σ_0_ indicates that the flaw population became broader rather than uniformly weaker, because failure in quasi-brittle polymers is governed by the most severe defect rather than by the average material response [15,17]. Water and pooled saliva probably produced more uniform sorption and plasticization, shifting the strength distribution without markedly increasing strength scatter [15]. In contrast, an active *S. mutans* biofilm may have generated a more localized challenge through esterase-mediated hydrolysis of methacrylate ester bonds and lactic acid production at colonized sites [11,21]. Surface and chemical changes were not measured directly in this study, so this mechanism is inferential; it is, however, consistent with reports that *S. mutans* and multi-species biofilms increase surface roughness and degrade resin surfaces and interfaces [4,6,8]. This localized biofilm-mediated challenge may have concentrated around surface porosities, polishing grooves, and, in printed resins, layer lines, thereby generating flaws of variable size. This interpretation is consistent with the Weibull results: strength scatter increased, as indicated by the reduction in m, while σ_0_ was largely preserved, indicating reduced reliability without a parallel loss of characteristic strength.

The performance of the printed resins was material- and environment-dependent, rather than consistently superior to that of conventional acrylic (Figure 1). In water, the two printed resins showed different behaviour relative to OC: BMC was more reliable and stronger, with an m value of 13.0 compared with 7.9 for OC, whereas KH showed similar strength to OC but a lower m value. Under biofilm exposure, this distinction was no longer evident, as the m values of all three materials converged to low values near 2. This suggests that the differences observed after water storage did not persist after microbial ageing, where localized degradation appeared to outweigh differences in the initial polymer network. BMC’s advantage in water may reflect a more controlled polymerization route, producing a more homogeneous and highly converted network than hand-mixed OC, which is more susceptible to variable powder-to-liquid ratios, entrapped air, and residual methyl methacrylate plasticization [22]. BMC was produced by digital light processing with standardized post-curing, which leads to a higher degree of conversion and a denser crosslinked network that restricts the free volume available for water ingress [23,24]. These findings suggest that the manufacturing route influences the initial mechanical performance of appliance polymers but may have a reduced impact once degradation is dominated by microbial ageing. A comparable pattern—similar mean strength but lower, more variable reliability in additively manufactured material—has been reported for printed versus milled zirconia [13,14].

The strength at the 5% failure probability (σ_5_%) quantified the lower-tail risk relevant to early clinical failure [17]. A three-parameter model was required for some groups, because a non-zero threshold strength (σ_μ_), the stress below which failure is not expected, arose from two unrelated mechanisms. In KH, σ_μ_ was present across all environments (28.9 to 38.7 MPa) with a uniformly low modulus, consistent with a more compliant methacrylate-based splint formulation. Such behavior may reflect greater tolerance for flaws, since compliant polymer networks can undergo crack-tip deformation and blunting, thereby reducing the severity of sharp defects rather than eliminating them [25,26]. In OC and BMC, σ_μ_ appeared only after biofilm ageing (near 41 MPa) and may be instead attributed to localized bio-erosion that reshaped the flaw population. Because the same feature arose by two routes, no single fixed model fitted the set, and model selection used the AIC, adopting the three-parameter model only when it gave a strictly lower AIC, rather than visual fit (Figure 4A). The fitted threshold also set a floor under the lower tail, which is why σ_5_% did not track the reduced modulus under biofilm. Forcing a two-parameter fit on KH biofilm underestimated σ_5_% by approximately 10 MPa (Figure 4B,C), holding a clinically viable resin to an unduly severe standard [17,27]. The three-parameter model should nonetheless be used with caution: the threshold σ_μ_ is sensitive to sample size and to the convention of fixing it near the lowest measured strength; it may overfit, and it carries two physical meanings, so it should not be read as a safe design stress.

This study has limitations. The biofilm was a single-species *S. mutans* model and therefore did not reproduce the ecological complexity of the intraoral biofilm [12]. The exposure was limited to 24 h, which corresponds to established early *S. mutans* biofilm formation (24–48 h) and was kept short deliberately, since longer incubations would require additional culture cycles whose variability could itself inflate strength scatter; nonetheless, 24 h may not capture long-term degradation. No masticatory or fatigue (cyclic) loading was simulated, and because the study assessed short-term static flexural strength, the in vitro reliability should be interpreted as an indicator of relative performance rather than a direct predictor of clinical failure, which also depends on fatigue, loading, and appliance geometry. Surface roughness, biomass, lactic acid production, local pH, residual monomer release, sorption, and degree of conversion were not measured; therefore, the proposed mechanisms and threshold values should be interpreted as approximate. Longer-term studies using multispecies biofilms and tracking subcritical crack growth are needed to better reproduce the time-dependent stress corrosion that governs clinical lifespan [12,16,28].

## 5. Conclusions

Exposure to different oral-relevant environments altered the reliability of appliance resins in a way that average strength alone could not capture. Microbial biofilm exposure primarily reduced the predictability of strength, as indicated by lower Weibull modulus values, rather than causing a proportional loss of characteristic strength. This indicates that conventional strength thresholds may be insufficient to qualify removable appliance resins when used alone and should be complemented by reliability analysis under biologically relevant conditions. For short-term or interim appliances, the differences among these resins are small and material choice is less critical, whereas a material whose modulus collapses after bio-ageing warrants caution; we do not propose a fixed minimum modulus, because no universally validated value exists. The response to bio-aging was not uniform across materials, showing that 3D-printed resins cannot be assumed to be inherently superior or inferior to conventional acrylic. Instead, their performance depends on how each formulation responds to the environment. Reliability should therefore be assessed using a Weibull model that reflects the observed strength distribution rather than applying a fixed criterion to all materials. These conclusions are based on biaxial flexural (static) testing and may not extend to other failure modes such as fatigue, impact, or wear.

## Figures and Tables

**Table 1:** Weibull parameters (Weibull modulus m; characteristic strength σ_0_; threshold strength σ_u_; and strength at 5% failure probability σ_5_%) for each material and ageing condition, with 95% confidence intervals from 2000 bootstrap resamples. The selected model (two- or three-parameter) is indicated; σ_u_ is reported only for three-parameter groups.

Group	Model	m (95% CI)	σ_0_, MPa (95% CI)	σ_μ_, MPa (95% CI)	σ_5_%, MPa (95% CI)
OC biofilm	3-param	1.95 (0.6–10.3)	55.4 (51.4–58.1)	41.1 (9.4–44.2)	44.3 (42.7–46.7)
OC saliva	2-param	10.01 (8.0–13.7)	57.3 (54.9–59.2)	—	42.6 (38.5–46.9)
OC water	2-param	7.98 (6.7–10.6)	54.8 (52.2–57.2)	—	37.8 (34.0–42.7)
BMC biofilm	3-param	2.62 (0.7–4.0)	63.2 (59.6–65.9)	41.5 (34.5–53.8)	48.5 (46.2–54.3)
BMC saliva	2-param	17.61 (14.8–23.2)	64.8 (63.4–66.0)	—	54.8 (52.6–57.4)
BMC water	2-param	13.01 (11.0–16.8)	65.4 (63.4–67.1)	—	52.0 (49.1–55.6)
KH biofilm	3-param	1.43 (0.6–2.5)	52.3 (48.1–55.3)	38.7 (36.0–42.9)	40.4 (39.1–43.5)
KH saliva	3-param	2.55 (1.4–7.8)	47.5 (45.4–49.5)	32.8 (16.5–39.6)	37.4 (36.0–40.7)
KH water	3-param	3.34 (0.7–6.5)	52.1 (48.4–54.4)	28.9 (12.9–42.7)	38.5 (35.6–43.7)

## Data Availability

The data presented in this study are available from the corresponding author upon reasonable request.

## References

[1] Topçu S., Tekçe N., Kopuz D., Özcelik E.Y., Kolaylı F., Tuncer S., Demirci M. (2024). Effect of surface roughness and biofilm formation on the color properties of resin-infiltrated ceramic and lithium disilicate glass-ceramic CAD-CAM materials. J. Prosthet. Dent..

[2] Haghi H.V., Peeri-Dogaheh H., Fazlalizadeh S., Abazari M., Mohammadhosseini R. (2021). Effect of *Streptococcus mutans* on the flexural strength of resin-based restorative materials. Dent. Res. J..

[3] Zhou X., Wang S., Peng X., Hu Y., Ren B., Li M., Hao L., Feng M., Cheng L., Zhou X. (2018). Effects of water and microbial-based aging on the performance of three dental restorative materials. J. Mech. Behav. Biomed. Mater..

[4] Fúcio S.B.P., Carvalho F.G., Sobrinho L.C., Sinhoreti M.A.C., Puppin-Rontani R.M. (2008). The influence of 30-day-old *Streptococcus mutans* biofilm on the surface of esthetic restorative materials—An in vitro study. J. Dent..

[5] Flemming H.-C. (1998). Relevance of biofilms for the biodeterioration of surfaces of polymeric materials. Polym. Degrad. Stab..

[6] Kusuma Yulianto H.D., Rinastiti M., Cune M.S., de Haan-Visser W., Atema-Smit J., Busscher H.J., van der Mei H.C. (2019). Biofilm composition and composite degradation during intra-oral wear. Dent. Mater..

[7] Zhang R.-J., Zhao L., Yu L.-X., Tan F.-B. (2024). Influence of thermal-cycling or staining medium on the surface properties and color stability of conventional, milled, and 3D-printed base materials. Sci. Rep..

[8] Li Y., Carrera C., Chen R., Li J., Lenton P.A., Rudney J.D., Jones R.S., Aparicio C., Fok A. (2014). Degradation in the dentin–composite interface subjected to multi-species biofilm challenges. Acta Biomater..

[9] Maleki T., Meinen J., Coldea A., Reymus M., Edelhoff D., Stawarczyk B. (2024). Mechanical and physical properties of splint materials for oral appliances produced by additive, subtractive and conventional manufacturing. Dent. Mater..

[10] Raffaini J.C., Soares E.J., de Lima Oliveira R.F., Vivanco R.G., Amorim A.A., Pereira A.L.C., Pires-de-Souza F.C.P. (2023). Effect of artificial aging on mechanical and physical properties of CAD-CAM PMMA resins for occlusal splints. J. Adv. Prosthodont..

[11] Huang B., Siqueira W.L., Cvitkovitch D.G., Finer Y. (2018). Esterase from a cariogenic bacterium hydrolyzes dental resins. Acta Biomater..

[12] Lohbauer U., Belli R., Ferracane J.L. (2013). Factors involved in mechanical fatigue degradation of dental resin composites. J. Dent. Res..

[13] Bergler M., König A., Korostoff J., Mante F.K., Hahnel S., Rosentritt M. (2026). Comparison of the flexural strength and microstructure of 3D printed and milled mono- and multilayer zirconia. J. Mech. Behav. Biomed. Mater..

[14] Pinelli L.A.P., Ferreira I., dos Reis A.C. (2025). Analysis of flexural strength and Weibull modulus of printed and milled zirconia: A systematic review. J. Prosthet. Dent..

[15] Kelly J.R. (1995). Perspectives on strength. Dent. Mater..

[16] Lohbauer U., Krämer N., Petschelt A., Frankenberger R. (2008). Correlation of in vitro fatigue data and in vivo clinical performance of a glassceramic material. Dent. Mater..

[17] Lim K., Yap A.U.J., Agarwalla S.V., Tan K.B.-C., Rosa V. (2016). Reliability, failure probability, and strength of resin-based materials for CAD/CAM restorations. J. Appl. Oral Sci..

[18] Morin J.L.P., Dubey N., Luong-Van E.K., Yu B., Sabino C.F., Silikas N., Agarwalla S.V., Castro Neto A.H., Rosa V. (2025). Graphene nanocoating on titanium maintains structural and antibiofilm properties post-sterilization. Dent. Mater..

[19] (2015). Dentistry—Ceramic Materials.

[20] Quinn J.B., Quinn G.D. (2010). A practical and systematic review of Weibull statistics for reporting strengths of dental materials. Dent. Mater..

[21] Huang B., Sadeghinejad L., Adebayo O.I.A., Ma D., Xiao Y., Siqueira W.L., Cvitkovitch D.G., Finer Y. (2018). Gene expression and protein synthesis of esterase from *Streptococcus mutans* are affected by biodegradation by-product from methacrylate resin composites and adhesives. Acta Biomater..

[22] Kostić M., Stanojević J., Tačić A., Gligorijević N., Nikolić L., Nikolić V., Igić M., Vasić M.B. (2020). Determination of residual monomer content in dental acrylic polymers and effect after tissues implantation. Biotechnol. Biotechnol. Equip..

[23] Kirby S., Pesun I., Nowakowski A., França R. (2024). Effect of different post-curing methods on the degree of conversion of 3D-printed resin for models in dentistry. Polymers.

[24] Chaudhary R., Fabbri P., Leoni E., Mazzanti F., Akbari R., Antonini C. (2022). Additive manufacturing by digital light processing: A review. Prog. Addit. Manuf..

[25] Liu B., Yin T., Zhu J., Zhao D., Yu H., Qu S., Yang W. (2023). Tough and fatigue-resistant polymer networks by crack tip softening. Proc. Natl. Acad. Sci. USA.

[26] Gehling T., Schieppati J., Balasooriya W., Kerschbaumer R.C., Pinter G. (2023). Fatigue behavior of elastomeric components: A review of the key factors of rubber formulation, manufacturing, and service conditions. Polym. Rev..

[27] Agarwalla S.V., Malhotra R., Rosa V. (2019). Translucency, hardness and strength parameters of PMMA resin containing graphene-like material for CAD/CAM restorations. J. Mech. Behav. Biomed. Mater..

[28] Belli R., Petschelt A., Lohbauer U. (2014). Are linear elastic material properties relevant predictors of the cyclic fatigue resistance of dental resin composites?. Dent. Mater..

